# Day time, night time, over time: geographic and temporal uncertainty when linking event and contextual data

**DOI:** 10.1186/s12940-021-00734-x

**Published:** 2021-05-04

**Authors:** David C. Folch, Christopher S. Fowler, Levon Mikaelian

**Affiliations:** 1 Department of Geography, Planning and Recreation, Northern Arizona University, PO Box 15015, Flagstaff, AZ 86011 USA; 2 Department of Geography, Penn State University, 302 Walker Building, University Park, PA 16801 USA; 3 Department of Geography, Florida State University, PO Box 3062190, Tallahassee, FL 32306 USA

**Keywords:** Uncertainty, Space-time, Neighborhood effects

## Abstract

**Background:**

The growth of geolocated data has opened the door to a wealth of new research opportunities in the health fields. One avenue of particular interest is the relationship between the spaces where people spend time and their health outcomes. This research model typically intersects individual data collected on a specific cohort with publicly available socioeconomic or environmental aggregate data. In spatial terms: individuals are represented as points on map at a particular time, and context is represented as polygons containing aggregated or modeled data from sampled observations. Uncertainty abounds in these kinds of complex representations.

**Methods:**

We present *four* sensitivity analysis approaches that interrogate the stability of spatial and temporal relationships between point and polygon data. *Positional accuracy* assesses the significance of assigning the point to the correct polygon. *Neighborhood size* investigates how the size of the context assumed to be relevant impacts observed results. *Life course* considers the impact of variation in contextual effects over time. *Time of day* recognizes that most people occupy different spaces throughout the day, and that exposure is not simply a function residential location. We use eight years of point data from a longitudinal study of children living in rural Pennsylvania and North Carolina and eight years of air pollution and population data presented at 0.5 mile (0.805 km) grid cells. We first identify the challenges faced for research attempting to match individual outcomes to contextual effects, then present methods for estimating the effect this uncertainty could introduce into an analysis and finally contextualize these measures as part of a larger framework on uncertainty analysis.

**Results:**

Spatial and temporal uncertainty is highly variable across the children within our cohort and the population in general. For our test datasets, we find greater uncertainty over the life course than in positional accuracy and neighborhood size. Time of day uncertainty is relatively low for these children.

**Conclusions:**

Spatial and temporal uncertainty should be considered for each individual in a study since the magnitude can vary considerably across observations. The underlying assumptions driving the source data play an important role in the level of measured uncertainty.

## Background

Geographic context matters when considering health outcomes. Physical location has been tied to obesity [1, 2], stress [3, 4], cancer [5, 6], stroke [7], dementia [8], and many other health conditions. Complicating matters is that context can be both generalizable and idiosyncratic in terms of contributing to the health outcomes of people whose lives intersect with a particular space at a particular time. Locations with high particulate matter (PM) are broadly harmful, especially to people who spend considerable time outdoors or are at elevated risk to PM. In contrast, lead pipes are generally not harmful as strategies have been developed to prevent lead from leaching into the water supply. For example, the 2014 Flint, Michigan water crisis was the result of public policy, changes in water sources, and aging infrastructure, among other factors; not simply the presence of lead pipes.

The role of space and time in health outcomes can be viewed through the three components of the classic epidemiological triad: host, agent, and environment. The host is the population at risk, the agent is what is causing harm and the environment is the milieu in which the two interact. While host and agent are reasonably straightforward concepts, *environment* has taken on many meanings that can include time, location, and interaction process. The importance of environment is clear, but its operationalization is opaque. Specifically, the interrelationship between the location and time *attributes* of the host and agent, and the space in which they interact is not straightforward.

These interrelationships are further complicated by uncertainty in the supporting data, which is frequently not considered. Uncertainty can arise through the quality of a global positioning system (GPS) or address geocoder used to convert places to latitude/longitude coordinates, through the choice of distance buffers used to define interaction spaces, or because the duration of exposure in a particular location is not well specified. These all fall into the opaque environment dimension of the triad, which does not always receive a high level of attention in research design. While patient interaction protocols and blood toxicity measurements are critical to research design and analysis, so too are the details of geolocation when environment is tested as contributing to a particular health outcome. The purpose of this paper is to illustrate both the mechanisms and magnitude of uncertainty related to geolocation in environmental health measurements. We further demonstrate how this uncertainty can be quantified and incorporated into assessments of the impacts of environment on health.

### Geographic uncertainty

Geographic uncertainty is an inescapable phenomenon [9], and there has been much work done to identify and deal with sources and causes of such uncertainty [10–13]. These problems have largely involved conceptual issues of spatial mismatch between the phenomenon under study and the available data.

The “uncertain geographic context problem” (UGCoP) was formalized by Kwan in [11, 14]. The *problem* is that researchers do not know the true spatial context that is relevant to a particular individual for a particular health outcome. Under this framework, the idea of contextual effects is assumed to be present, where context is some area containing people or environmental conditions associated with the health outcome. If the size and shape of that area is measured imprecisely, then the analytical results based on those shapes will also be imprecise. A second dimension of UGCoP is that individuals are not fixed in space, meaning that a person’s context changes throughout the day and over the life course. The ambiguity around time spent in various areas can again affect results.

The modifiable areal unit problem (MAUP) is somewhat parallel to UGCoP, and concerns the issue that some analytical results significantly change if the same overall study area is partitioned differently [10, 15]. Partitions can differ in terms of the number of areas and/or the locations of the dividing lines. The *problem* again is that the true spatial partition defining the relevant context for a particular environmental phenomenon as it applies to a particular individual is unknown.

More recently, Fowler [12] introduced the idea of “contextual fallacy.” The more widely known “ecological fallacy” concerns the problem of assuming that aggregate data is representative of any particular individual in a place [16]. Contextual fallacy concerns the problem of assuming that contextual data on place “adequately reflects the experiences of individuals” in that place [12]. It recognizes the growing literature in health, and social science more broadly, that oftentimes puts considerable weight on contextual data that can vary considerably across a region. People located at different places within some contextual area likely have different experiences; but the single context variable is assumed to be equally applicable to them all.

To a large extent, the UGCoP, MAUP, and contextual fallacy are warnings to researchers that just because data can be easily acquired, it may not be appropriate for a particular research question. Census tracts and block groups are defined using visible and political boundaries in the landscape, but they are not necessarily “neighborhoods.” Satellite imagery provides information about the entire planet in square pixels, but the real world is organic and rarely conforms to a regular raster. In principle, primary data collection can resolve many of these challenges by matching the data directly to the research question. However, primary data collection is not cost effective for many research activities, so the question remains: how well do the convenient datasets align with the research objectives?

### Connections to environmental health

The environmental health literature abounds with examples of techniques that recognize these sources of error in the link between individual and context and try to remedy them. The research on the links between health outcomes and exposure to pollution from automobile traffic is exemplary in this respect. Because the expected negative health effects of this mechanism diminish quickly with distance, precision is at a premium [17–22]. This literature has explicitly dealt with issues of positional accuracy related to geocoding [20, 23–27] as well as the significance of taking measurements only at place of residence [22]. While these studies demonstrate how significant precise geographical and spatio-temporal measurement are to health outcomes, they leverage detailed data sources such as activity diaries and orthographic photogrammetry to gain precision [17, 27]. While clearly superior, these data sources are not always available. In contrast, the literature contains numerous examples of studies where place of residence or hospital where treatment occurred stand in for an individual’s context; practices that introduce significant uncertainty into findings [28, 29].

Ultimately, few health researchers are also expert in issues related to geographic uncertainty. Neither the form that this uncertainty is likely to take nor its likely impact on analytical results is widely considered in most health research. When addressing issues in environmental health this problem is attenuated because of the complexity of methods involved in assigning contextual effects where both level of exposure and level of uptake require significant assumptions. Our aim here is not to add to the literature on the value of increased precision, but to demonstrate how sensitivity analyses of geographic uncertainty might be conducted. In so doing we wish to emphasize the degree to which careful consideration of spatio-temporal uncertainty is critical to the robustness of findings resulting from studies that link environmental context to individual health.

We consider four pathways that geographic uncertainty can enter into our assessment of environmental impacts, and we construct measures that allow us to quantify this uncertainty in general terms, and with respect to our specific population. Ultimately, the feasibility of our specific measures will be a function of the data available and the research question being asked. In the empirical example we use here, we uncover significant uncertainty resulting from assumptions built into the model determining the spatial distribution of toxicity; model assumptions we might not have otherwise been aware of. The spatial patterning of this uncertainty is, itself, an area of concern as it may introduce bias into the quality of our findings that is non-random in nature but subject to a complex geographic pattern that is not easily accounted for.

## Methods

We demonstrate the impact of uncertainty in four spatial and temporal inputs to the research process using a sensitivity analysis approach. The first is positional accuracy. A common scenario is the allocation of observations with a latitude/longitude attribute to some polygon, e.g., a census tract or zip code; this case assesses the significance of getting this assignment process correct. The second case is neighborhood size, which is typically the size of the area in which interactions are assumed to be relevant. Because *neighborhood* may be defined differently in different research contexts and may ultimately be chosen based on data availability rather than theory or empirical analysis, it is important to understand how size may impact observed results. Third is life course. Data in environmental health contexts are often a snapshot of an individual at some point in time; shift the analysis a few days, months, or years and the results might differ. When impacts are assumed to be based on exposure, how much do we need to understand about the consistency of exposure over time in particular places or the propensity of individuals to move, thus potentially altering their exposure over time? The fourth case is closely related to the third by considering time of day rather than longer temporal change. People tend to occupy different spaces throughout the day; exposure is not simply a function of where people live, but all the locations where they spend time. Since a significant portion of administrative data is reported based on place of residence, what impacts might changing context over the day introduce into our understanding of exposure? Our efforts in each of these cases are focused on introducing the mechanisms through which a specific type of spatio-temporal uncertainty can enter an analysis and offering a tool to quantify the magnitude of this uncertainty. These tools are not intended to replace more precise attributions of contextual effects to individuals, but to provide opportunities for sensitivity analysis when more precise measurement is not feasible.

We study these issues by intersecting two longitudinal and georeferenced datasets: the Family Life Project’s (FLP) point data on a specific cohort of children and the US Environmental Protection Agency’s (EPA) Risk-Screening Environmental Indicators (RSEI) model that provides area data on air toxicity.

### Point data

FLP data offers longitudinal health information on a representative sample of *N* = 1292 children, and their primary caregivers, who were born over a one-year period in 2003 and 2004 in three low-income rural counties in Pennsylvania and three in North Carolina.^1^ The data collection phase continues today, but we focus here on the 8 contacts starting at 2 months and ending at 60 months. Even as the children moved, all but a few contacts in the entire dataset were done in-person. The present analysis focuses on Pennsylvania and North Carolina, and so excludes those contacts that occurred outside of these states. The interview protocol involved documenting the address and GPS coordinates at each visit [30]. Contacts were made at both the child’s residence and day care location (if different from their residential location).

The combination of self-reported addresses, addresses reported by data collectors, and GPS readings allowed for cross-validation of the physical location reported with each contact in the FLP data. The cross-validation step helped address a number of challenges in the dataset: non-functioning GPS at time of visit, addresses that could not be accurately geocoded, and typos in the transcribed data. FLP is unusual for the thoroughness with which location information is handled; this allowed most location issues to be mitigated through the multiple data sources. Data collected with fewer methods of corroboration should be expected to introduce more uncertainty, which is partial motivation for the analysis that follows. The final locations used in the analysis are those that could be corroborated by two sources. Table 1 shows the distribution of visits by age, state, and location type, for a total of 13,973 data points.

**Table 1:** Counts for FLP sample

	Day Care		Residence		Total	
Visit Mo.	NC	PA	NC	PA	NC	PA
2	599	378	636	400	1235	778
6	597	377	611	403	1208	780
15	635	432	662	453	1297	885
24	631	421	655	455	1286	876
35	626	400	654	442	1280	842
48	518	330	596	409	1114	739
58/60	418	225	607	403	1025	628
**Total**	**4024**	**2563**	**4421**	**2965**	**8445**	**5528**

### Context data

The toxicity data is built by EPA from its Toxic Release Inventory (TRI) database. TRI was initiated in 1986 and now collects data on releases of over 675 toxic chemicals from over 22,000 industrial facilities across the country [31]. The RSEI model translates those point level air and water releases into toxicity “scores” for 0.5 mile (0.805 km) cells for the entire country. RSEI integrates quantities of toxins from the TRI database with measures of hazard and risk to generate unitless toxicity scores. RSEI also uses environmental conditions such as topography and wind direction along with site conditions such as emissions stack heights to develop a distance decay factor that associates the impact of a release to surrounding pixels up to 30 miles (48.280 km) away. Therefore, a pixel’s score reflects the total toxicity in that pixel not just the impact of releases that happened in that pixel. A key strength of the RSEI design is that scores for individual releases are standardized in such a way that they can be summed across time and toxin to get aggregate toxicity scores even when the nature of the toxicity and its presumed effect on health can vary immensely. Furthermore, the data, including historic data, are regularly updated as new information is made available about the toxicity of particular substances. This makes it relatively easy to compare toxicity scores over time even when our understanding of effects may improve with new science. A downside of the RSEI approach is that different toxins will have different release types (e.g., short-term high-volume vs. long-term low-volume) and different dispersion patterns that nevertheless get combined into the same annual measure of toxicity. These different types of release would suggest different pathways for individual uptake thereby introducing another form of spatio-temporal uncertainty that we do not address here.

There are 201,457 pixels in North Carolina and 181,805 in Pennsylvania. For the purposes of this analysis we rely on the total toxicity score; summed across all releases in a given year. Parallel work considers specific toxins individually [32]. The EPA's 2018 report [33] contains full details on the EPA’s methodology.

The annualized RSEI data provides a toxicity score and estimated population for each pixel for each year. RSEI pixel data was then linked to the FLP children’s point locations (residential and day care) based on the pixel in which the point fell and the date of the visit; this provides environmental context for the FLP children through time. With this data we can explore spatial and temporal patterns in environmental toxicity for the statewide context (i.e., all pixels in the state averaged), the statewide population (i.e., population weighted pixel scores), and the FLP population.

**Fig. 1:**
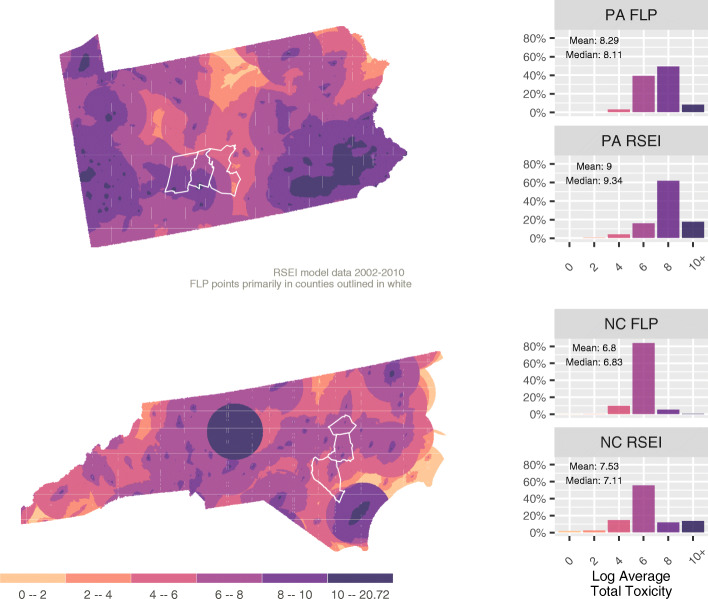
Log of Average Total Toxicity

Each RSEI pixel is 0.25 mile^2^ (0.647 km^2^), but the population in each pixel varies from 0 to 4413.41 in North Carolina and 0 to 12,352.4 in Pennsylvania; 9 and 5.2% of the pixels respectively have zero population, reflecting the rural nature of large portions of the states (considering all pixel-years). RSEI context scores range from 0 to 2,338,496,000 in North Carolina and 0 to 54,603,400 in Pennsylvania, both with long tails (considering all pixel-years). The correlation between population counts and logged toxicity context by pixel ranges from 0.1098 (2003) to 0.1695 (2005) in North Carolina and between 0.1902 (2009) to 0.2206 (2004) in Pennsylvania. These correlations indicate a relatively low correlation between population and toxicity in both states indicating that rural areas with lower population are potentially as exposed to toxicity as those in urban areas. When comparing the FLP population in particular to the distribution of toxicity as a whole, the FLP population has a significantly lower average total toxicity than the respective state populations (shown as the log of average total toxicity in the bar charts in Fig. 1).

The spatial variation in the toxicity values is presented in the maps in Fig. 1 as the average annual toxicity by pixel. Pennsylvania has a clear pattern of higher toxicity in the West and Southeast, with concentrations primarily around Pittsburgh and Philadelphia. Toxicity tends to decline toward central Pennsylvania, but high pockets are scattered throughout the state. Of note is that the FLP households in Pennsylvania (primarily in Blair, Cambria, and Huntingdon Counties in south-central Pennsylvania) are located in a relatively high toxicity area even if the individual observations tend to be at or below the norm for the State. North Carolina has two concentrations of toxicity in the Winston Salem and Wilmington areas each seemingly driven by a single release site (based on the clear circular pattern expressed on the map). There are also scattered areas of high toxicity throughout the state. In terms of average total toxicity, the North Carolina FLP households (primarily in Sampson, Wayne, and Wilson Counties in east-central North Carolina) tend to be in slightly lower toxicity areas than Pennsylvania FLP households.

In both North Carolina and Pennsylvania the FLP population is located outside of the areas with the very highest categories of average annual toxicity. Delving deeper into the annual data, FLP populations are occasionally located near major toxicity events, but when considering the population across all years they tend to avoid the most extreme areas of prolonged exposure.

RSEI model limitations are clearly visible in the maps, especially for North Carolina. The model’s 30 mile (48.280 km) maximum effect radius appears as a series of circles and semi-circles that represent significant, and empirically unlikely, drop offs in toxicity exposure. These drop offs are visible even when considering the log of the RSEI toxicity score and point to the intensity of effects attributed to a small number of acute events and the outsized role these events play in shaping overall model outcomes. Despite these limitations, the RSEI model remains one of the only products available to estimate airborne toxicity exposure nation-wide, over multiple years, and with a reasonable level of geographic specificity.

## Results

In this analysis we explore four forms of geographic and temporal uncertainty. We begin by investigating the importance that a person is geolocated to the correct cell. Next, we address the spatial scale of our assessment of toxicity exposure by considering various neighborhood sizes around each person. The temporal dimension in toxicity exposure first considers longitudinal change in exposure over years, looking separately at people who remain in the same place and those who move. Finally, we study cyclical time in the form of daytime versus nighttime exposure. In most cases we are able to compare the statewide population to the FLP population; the temporal cases highlight the value of the FLP’s data collection model for these tests.

In each of the following sub-sections we document both the method used to apprehend geographic uncertainty and the results of that method applied to the FLP and RSEI data. The forms of geographic uncertainty present in our data emerge out of a data structure that seeks to match point-level information on individuals with gridded environmental data; a data structure relevant for many kinds of environmental health research. By combining specific methods and results in each sub-section we hope to emphasize the importance of the specific geographic problem being faced and the types of methodological solutions available to assess its magnitude.

Each measure presented examines the impact of uncertainty in the relationship between the geolocation of an individual within the dataset and their contextual data. They recognize that researchers may have to use data where accuracy is either of poor quality or where accuracy is unknown. When researchers cannot fix a problem it is still important to understand the degree to which that problem could impact downstream results. The methods in the sub-sections that follow allow for the quantification of uncertainty. In our concluding section we describe ways that researchers might use these measurements to test the robustness of their findings.

A note on representation in this section. The toxicity scores include many low values and a small number of extremely high values, which affects the visualization of results. In lieu of transforming the results into logarithms, we adopt a consistent set of 6 logical custom bins for all the measures. The first two bins, 0–0.025 and 0.025–0.05 characterize very low and low levels of uncertainty; the third, 0.05–0.75 is moderate uncertainty; and the final three bins are high and very high levels. The highest bin for all the figures is 5 and above; the upper end of this last bin varies for each measure and is presented in the map’s legend. This approach increases the ease with which we can compare magnitudes across measures, but it can obscure some nuances within each measure.

### Positional accuracy

Positional accuracy measures the importance of a point being located in the correct pixel. Depending on the method by which a point was acquired, accuracy can vary widely. Modern GPS units such as those built into phones can be accurate within ± 4 m, but that accuracy can shift depending on the presence of buildings and other obstacles. Incorrect use of cartographic projections, or even simple transcription errors can and do affect the accuracy of geolocated data. More common is geolocation based on geocoded addresses, which can vary widely in quality based on the degree to which an address matches information in the geocoder. The difference between a geocode matched to a rooftop and one based on interpolation of an address along a road segment may seem insignificant, but it can alter the location of a point enough to impact which pixel or administrative unit it is assigned to [34]. Even greater uncertainty emerges when location is assigned using only the zip code or city information in the address; these assignments give an appearance of exactness (the same number of significant digits are reported for the latitude and longitude) while masking tremendous uncertainty about where within the administrative unit an individual is actually located.

If a pixel and its neighbors are relatively similar, then being incorrectly placed in a neighboring pixel will not have much impact on the results of subsequent analyses. In contrast, high variation among neighboring pixels implies that positional accuracy is more important. We measure the level of uncertainty associated with positional accuracy using the positional coefficient of variation (PCV), which we define for pixel *i* as:

$$ PC{V}_i=S{D}_i^{neighborhood}/ es{t}_i, $$

where *est*_*i*_ is the estimate for pixel *i* and $S{D}_i^{neighborhood}$ is the standard deviation of the estimate for pixel *i* and its neighbors. As will be seen later, “neighborhood” can be defined in many ways. We define neighborhood here as *i* and its eight immediate neighbors. Pixels are 0.5 mile (0.805 km) on a side meaning this neighborhood of nine pixels is 1.5 miles (2.414 km) on a side. The data we are working with was generated based on GPS coordinates matched to geocoded address data, so the 1.5 mile (2.414 km) range of our neighborhood fully captures the level of uncertainty we have in our assigned point locations. A study with finer resolution environmental data or with point assignment based on administrative unit centroids might require different neighborhood extents than what we use here.

**Fig. 2:**
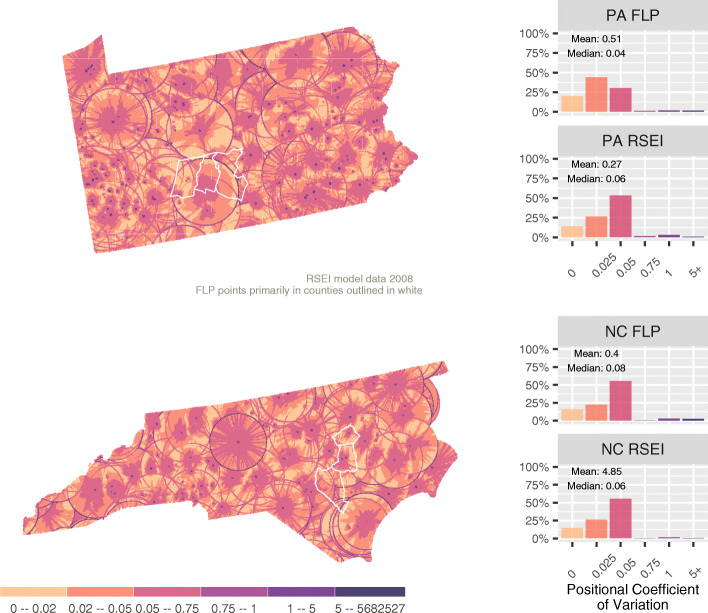
Positional Coefficient of Variation

The maps in Fig. 2 show PCV for 2008 (only one year of results is presented here) with lighter colors indicating lower PCV and thus less importance of small errors in a point’s location. In general, locations closer to a release have a high PCV, with the PCV decreasing with distance. In the RSEI model, toxicity is assumed to decrease rapidly, even exponentially, as distance increases from the release site. As a result pixels near major releases experience rapid change with distance and the precise placement of individuals in these cells will significantly impact reported contextual measures. Moreover, the RSEI model radius is noticeable in the maps; in this case as dark rings. The abrupt end to the estimated toxicity from an acute release can result in neighboring pixels on the edge of the 30 mile (48.280 km) radius from the event having extremely different values, and thus high PCV.

It is also possible to estimate the impact of PCV on all residents in the state and FLP homes in particular. The RSEI program estimates the number of people in each pixel based on US Census Bureau data, which allows us to sum people by PCV. We can further assign each FLP address to the pixel in which it falls to measure its PCV. These results are presented in Fig. 2 as population weighted histograms. Figure 2 has some interesting properties. First, while North Carolina and Pennsylvania are fairly different in terms of their toxicity context (Fig. 1), their statewide PCV distributions are remarkably similar. One explanation for this similarity is that the effect of uncertainty being measured in the PCV is likely conditional on model parameters that structure rates of decay and effects of distance in the RSEI model more than it is related to specific toxicity releases. Second, while PCV for North Carolina’s FLP sample diverges little from the state population (in distribution and median values), the Pennsylvania FLP sample has a noticeably different distribution and median skewed towards lower PCV than the population as a whole (although the mean is higher).

### Neighborhood size

The concept of ‘neighborhood’ is contested within social science research [35] because it has a sociological meaning, but it is rarely measured as an administrative unit for which data are collected and released. In many cases the neighborhood represents an important context in which we might theorize some relationship between environment and individual, but the precise measurement and definition of neighborhood depends on a unit of analysis that will be, at best, an approximation. Common neighborhood metrics in the U.S. context include census tracts, zip codes, and occasionally school districts. Research has shown that the size of the unit will condition what we observe as well as the variance among our observations [12]. Significantly, if we use smaller units to describe our neighborhood context we will observe more extreme results and more variation between units. Larger units will tend to offer less extreme results and lower variation due to smoothing. The implications of these choices can therefore be significant for many types of analysis.

Here we explore the implications of different assumptions about neighborhood size that might parallel decisions made about whether to use, for example, census tracts or zip codes as a measure of neighborhood. For airborne toxicity we would expect impacts from a release to be conditioned on distance from the release. The challenge is then to operationalize “neighborhood” as a relevant geographic area surrounding an individual’s location. If the neighborhood assigned is too big, the variation among cells will smooth to the mean eliminating variance among individuals. If the neighborhood is too small, then minor perturbations in the spatial pattern can have outsized effects, overstating the variance among individuals. This problem has been shown to be especially problematic with low probability events such as murder or some cancers where a single event could greatly spike the incidence for a small geographic area [36]. The TRI data, which underlies the RSEI data, has a similar issue where extreme toxicity events have an outsized impact the pixel values.

We measure the sensitivity of measured toxicity to neighborhood size by first computing some function for neighborhoods of different size around pixel *i*. The neighborhood coefficient of variation (NCV) is computed over these values as:

$$ NC{V}_i= SD\left(f\left( neighborhoo{d}_{i1}\right),f\left( neighborhoo{d}_{i2}\right),\dots, f\left( neighborhoo{d}_{is}\right)\right)/f(i). $$

**Fig. 3:**
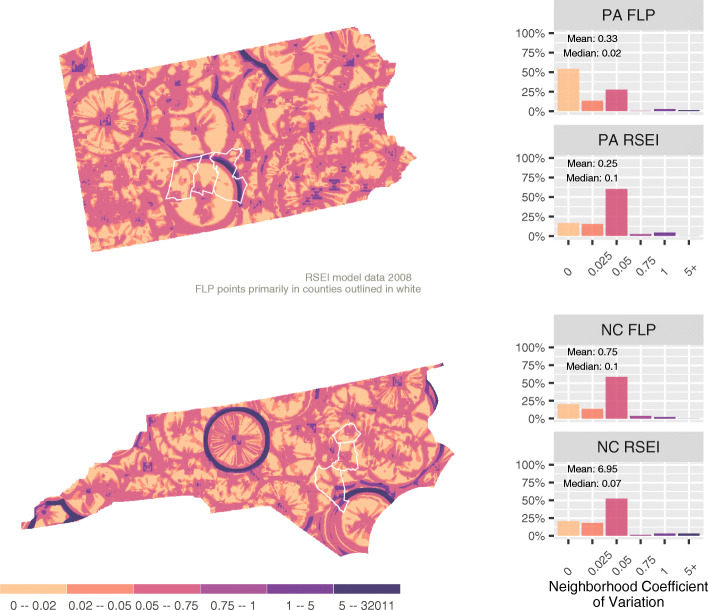
Neighborhood Coefficient of Variation

For the RSEI data, the function will be the average of the estimates in the neighborhood, where the neighborhoods are defined as square areas with the following numbers of pixels: 1, 9(3 × 3), 25(5 × 5), 49(7 × 7), 81(9 × 9), and 361(19 × 19). The resulting NCV values are presented in Fig. 3 for 2008. In light colored areas, there is little difference in the average toxicity as neighborhood size changes; darker areas indicate greater sensitivity to neighborhood size. Figure 3 displays a similar pattern to that for PCV of high value rings, but in contrast tends to have low values near the very center of the release sites. One feature that emerges in Fig. 3 is the role played by model assumptions that assume a very rapid decay effect within the first half mile (0.805 km). The maps show small 9 × 9 squares that emerge because the specific pixel where a given high toxicity release occurred will have a much higher toxicity than even its nearest neighbors. NCV calculations that just have that cell in their largest neighborhoods will have a much higher standard deviation and create the dark purple hollow boxes seen on the maps.

The state maps of NCV have two significant implications for uncertainty in assessing environmental health impacts. First, the generally low values for NCV across most of both states even when calculated across large neighborhood size differentials suggests that the rate of variation from cell to cell in the RSEI model is generally fairly low. While the RSEI model reports results at a relatively fine grain of 0.5 × 0.5 miles (0.805 × 0.805 km), the effective variation is mostly at much coarser scales. As a result, larger or smaller “neighborhoods” (at least as that term is employed in the social science literature) will largely produce the same result with this data. However, the spatial patterns that emerge in the NCV maps show that artifacts of assumptions made within the RSEI model play an outsized role in conditioning the variation in NCV that we observe. Put another way, the degree to which our assumptions about neighborhood size will matter is a function of assumptions about the form that distance decay takes in the original environmental impact model. This form of spatially structured uncertainty driven by model assumptions should act as a call for caution when linking environmental and health data sets together. The linkage among assumptions in different data sets can create complex forms of uncertainty in research results obtained with modeled data.

Second, in examining the histograms in Fig. 3 we can see that, while the Pennsylvania FLP sample embodies less uncertainty based on neighborhood size than Pennsylvania residents more generally, the reverse is true for North Carolina’s FLP population. Consequently, we can conclude that decisions about neighborhood size in subsequent work will impact results more strongly in the North Carolina sample than they will in the Pennsylvania sample. While NCV is relatively small in most places, it represents a significant effect in a small portion of the observations.

### Life course

By necessity, much of the research in environmental health will be cross-sectional. A significant portion of this research will also require assumptions about duration or consistency of exposure to a particular environment or contextual factor. Uncertainty may arise when it is unclear how much the context in a particular location changes over time, or how the location of a particular individual changes over time. Our assessment of life course uncertainty captures change in residential context over time.

Change in context can happen via two channels: 1) a person stays in the same home as the neighborhood transitions around them and 2) a person moves homes resulting in a change in context. It is important to note that neighborhoods tend to be stable, so channel 1 change will likely happen slowly. In contrast, a residential move offers the *opportunity* for a large shift in residential context, but the family could move to a statistically similar location. We therefore define life course coefficient of variation (LCV) for pixel *i* based on the standard deviation of toxicity scores over *n* time periods divided by the median of those *n* scores:

$$ LC{V}_i= SD\left( es{t}_{i1}, es{t}_{i2},\dots, es{t}_{in}\right)/ median\left( es{t}_{i1}, es{t}_{i2},\dots, es{t}_{in}\right). $$

**Fig. 4:**
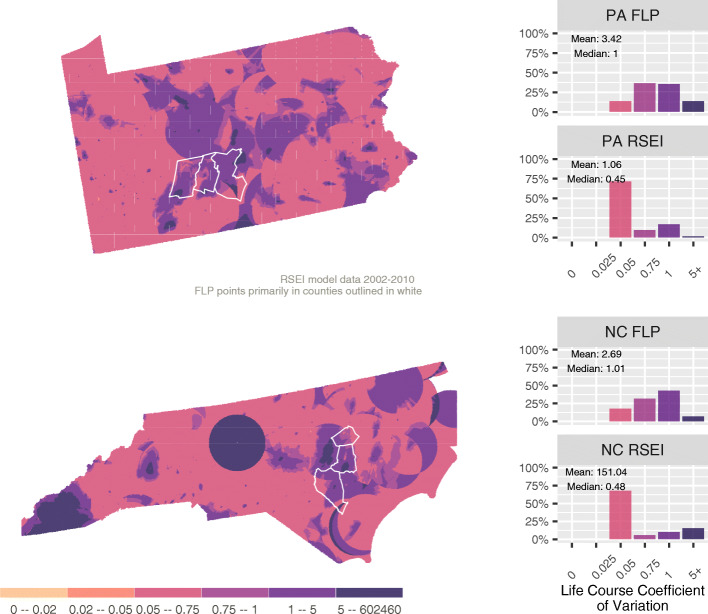
Life Course Coefficient of Variation

Figure 4 shows that the LCV is generally higher than the previously mapped coefficients of variation. This suggests that the timing of cross-sectional analyses may be strongly related to observed outcomes. This is, perhaps, not entirely surprising when considering a data set based off of event data like that in the TRI. The TRI data is characterized by extreme values, but given the cross-sectional nature of so much environmental impact research it serves as an important reminder. While we often conceive of neighborhoods or places as being polluted (or not), how those places appear in terms of measured toxicity can vary significantly from year to year.

LCV gives us a sense of how important the year we choose for assessment of environmental toxicity will be in terms of conditioning the results we observe. It is important to note that the maps and statewide histograms in Fig. 4 only reflect channel 1 type change since we do not have information on residential moves in the statewide dataset. However, the stability of toxicity in a place is only one part of the story, we also need to consider the stability of the population in a particular place (channel 2 type change). The FLP data give us this opportunity, which is to explore the effects of residential movement on our population. For the FLP families we know their locations at different points in time; this gives multiple possible transitions for each family and clearly identifies where a family moved. This is reflected in the histograms in Fig. 4 where the statewide histograms are relatively similar, and quite different from the the FLP histograms. The FLP distributions reflect more high values, and the median and mean values are higher since they are eligible for channel 1 and 2 changes.

**Fig. 5:**
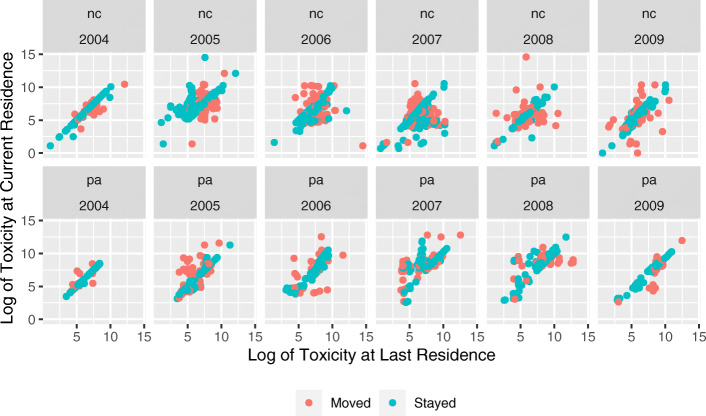
Experience of toxicity compared to previous years by whether household moved that year

With the FLP data we can more closely examine how the environmental context differed for families that stayed in place as opposed to families that moved. Figure 5 compares the toxicity from the previous year to the toxicity in the present year for FLP families that moved and families that stayed in the same residence that year. The figure shows a strong correlation year over year for stayers. Across all years the correlation was 0.73 in North Carolina and 0.81 for Pennsylvanians. Year over year correlations were significant but much lower for families that moved in a given year; 0.39 for North Carolina and 0.66 for Pennsylvania. The important differences between states, but most importantly between households that moved and households that stayed in the same residence gives a sense of the magnitude of uncertainty introduced when no information is available about the life course of individuals’ presumed exposure to a particular contextual effect. Notably, the direction of the effect of a move on toxicity level does not appear to be significant, with households almost equally as likely to increase their toxicity exposure in a new location as to decrease their exposure.

### Day-night

The fact that most administrative data counts individuals at their place of residence elides the importance of daytime location as an important factor determining exposure. Day-night uncertainty is similar to life course in that it accounts for locational variability over time, in this case over a single day. For working adults this is typically work and home, for older children it is school and home, and for young children it is day care and home. A substantial literature has begun to address this issue with regards to adults and the workplace [37]. Another literature asks similar questions about differences in context for children at home and at school [38]. Here we explore differences for children before they enter school, an area that has not been widely studied. We expect that differences between home and day care locations will be relatively small as child care will tend to be tightly constrained by both parents’ income and the need to remain relatively close to very small children. Nevertheless, the FLP data gives us an opportunity to test this hypothesis with respect to toxicity exposure.

**Fig. 6:**
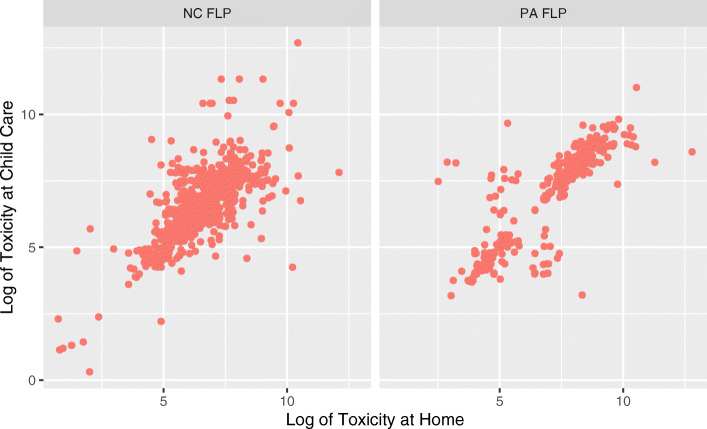
Comparison of Daytime and Nighttime Toxicity Exposure

Since we do not have day-night data on the statewide population, we only conduct this analysis on the FLP children based on their home and child care locations. In this case, each observation is a point in time where we have contemporaneous home and child care location information.  Figure 6 shows unequivocally that, for our dataset, the difference in exposure between day time care and night time residence is minimal. Across all years the correlation was 0.79 in North Carolina and 0.89 for Pennsylvania.

We draw two conclusions from these findings. First, we would expect parents to limit the distance between residence and child care facility for a variety of reasons, which reduces variation between daytime and night time exposure. Nevertheless, we also see some of this correlation as a function of the relatively low spatial variation in toxicity highlighted in both PCV and NCV maps; a model with higher spatial resolution might find more difference than we express here. Combined, the sensitivity analysis indicates that a single location is probably appropriate for this population and context data. This supports an argument to include observations missing home or daycare location information in subsequent toxicity analyses, as opposed to dropping them as incomplete.

## Discussion

Location and time have become common attributes of health datasets. Many tools are available to convert participant addresses into latitude/longitude coordinates, and nearly every phone can report its coordinates using a built-in global positioning system (GPS). Geographic information systems (GIS) are becoming more accessible and user friendly, which is further unlocking spatial analysis tools for a wide audience.

In contrast to the current ease of collecting an individual’s space-time data, individualized exposure data remains elusive. As a person moves through their day (and life), minor variations in their environment will affect the magnitude of their exposure to pollutants. The ideal approach is then to measure a person’s microenvironment using a portable sensor that passively captures the characteristics of their immediate surroundings [39]. Portable sensors exist to measure air pollution [39], radio frequency electromagnetic fields [40], noise pollution [41] and other harmful characteristics of the environment, but these need to be issued to participants and become an extra thing the person must carry around. In addition, there are no sensors for socioeconomic and other types of important microenvironment exposures. Ecological momentary assessment surveys are another microenvironment data collection strategy. These query a participant, typically via their smartphone, at defined intervals or when their device enters certain spaces as identified by the GPS. The participant can be asked to record any number of contextual variables based on their observation or by activating a sensor. These systems require careful design [42] and require active engagement from the participant. Myriad reasons preclude widespread precise space-time tracking of microenvironments, which leads to hybrid approaches that approximate this ideal data. In particular, a person’s exposure can be approximated by intersecting their locations with data on the context of areas. The mixture of precise and generalized datasets, and the assumptions grounding their commingling, are complex and demand further examination.

The sensitivity analysis methods presented in the previous section focus on a specific but common case in health research: the researcher has a cohort of individuals being investigated and is interested in the role of context on the subjects. The researcher typically has extensive and specific data on each individual’s attributes since the researcher is in control of the data collection protocol. In contrast, the context data is generalized or modeled from samples collected by someone else and is typically provided by a governmental agency as polygons. The implicit goal is to develop new knowledge by intersecting the deep understanding the researcher has of her own cohort with data developed by domain experts in entirely different fields. While this juxtaposition of expertise can lead to groundbreaking insights, there are also potential pitfalls.

The assumptions driving environmental, demographic, and other datasets can have non-trivial impact on the data and analyses that depend on them. The RSEI model, for example, integrates data on toxic releases, toxicity of chemicals, and weather patterns, to name a few inputs, to ultimately generate a detailed nationwide dataset on air pollution exposure. Demographic data is also complicated in its sampling strategies, weighting techniques, and imputations [43]. Location is embedded in many of these assumptions. For example, the previous section highlights the impact of the 30 mile (48.280 km) radius assumption in the RSEI data. In demographic data, the smaller the polygon the fewer samples available to support the estimates. This is an issue faced by the American Community Survey, which sees large increases in uncertainty as polygon size is reduced [44].

One approach to reducing the potential problems identified in the previous section is to increase the size of the polygons. The larger the polygon, the less likely an error of say 0.75 miles (1.207 km) would land the point in the “wrong” polygon. Similarly, larger polygons are more likely to capture a person’s home and work locations. This even extends to residential moves. According the US Census Bureau’s Current Population Survey, 64% of people who moved between 2017 and 2018 remained in the same county. While larger polygons potentially solve one problem, there is often a desire to use the smallest possible polygon in order to reasonably represent the interactions being studied. Crime, for example, varies greatly across most counties; therefore, the county average crime rate is unlikely to be representative of the likelihood any individual has of experiencing crime in their daily life. Therefore, there is no simple rule of thumb such as “use the largest (smallest) unit available.”

Since researchers are usually relying on secondary context data, they are forced to adapt their research design to the best available data. In terms of spatial scale, it is unlikely that the spatial units provided by some other entity will exactly match the research question(s) at hand. That being said, there is a continuum of spatial correspondence. On one end of the spectrum are clearly defined spatial units. For example, when access to particular treatments depends on the offerings of the county health department, then county of residence is a clear factor for the individuals being studied. The other end of the spectrum is context data provided as points. For example, air pollution monitoring station readings, Superfund sites, or crime incidents give the researcher the ultimate freedom to define the context for each of their participants. Most researchers use data that falls between these two extremes for very practical reasons: we are rarely lucky enough to have research relevant polygons and point data is typically too raw for non-experts to work with.

These challenges can be partially addressed by taking a critical view of geolocated data and seeking verification and validation of location information in the ways suggested here. Researchers must always seek to understand the cohort to understand how meaningful any single measure of location is likely to be with respect to the contextual phenomenon under consideration. Most geocoders provide a reliability estimate along with the latitude/longitude coordinates that can help with the exactness of coordinates. More broadly, collecting data on previous residence, place of work (school, day care, etc.), and time living at the location can provide important information about the significance of geolocated values. Similarly, context measures often have their own built in reliability measures that are overlooked far too frequently. Many public datasets come with measures of reliability such as margins of error (MOE). Failure to consider the range of contextual values that could fall within margins of error increases the supposed certainty of relationships while potentially introducing noise that can problematically shape the interpretation of outcomes.

In this paper we have addressed spatial uncertainty and spatio-temporal uncertainty but our approach is not comprehensive particularly with respect to the complications related to time and exposure. For example, the RSEI data is built up from the EPA’s Toxic Release Inventory, a database of events, but it reports outcomes in annual terms when much of the reported toxicity may have been extant in the air for only short periods of time. If an FLP household happened to be out of town (or inside with the windows closed) during a major release they might have a radically different exposure than their immediate neighbors. This mismatch is related to our motivation for examining NCV and LCV, but the structure of this temporal variation is both significant and poorly represented in our measures here. As a result we intend for our presentation here to motivate researchers to look for sources of uncertainty when linking individual and contextual information, rather than to define the universe of ways in which these data types might interact.

## Conclusion

The methods presented here are not an exhaustive set of metrics, they are exemplars of a broad sensitivity analysis approach to spatio-temporal uncertainty. The impact of any sensitivity analyses requires a carefully formulated question and set of tests to observe. In our case we required methods that intersect air pollution exposure data available as polygons and home and day care locations available as points. This polygon to point relationship is common in health and environmental research, meaning that these methods are useful for a many specific research questions. In particular, our measures (PCV, NCV, and LCV) do not rely on any specific domain expertise on environmental toxicity in order to estimate the structure of geographic and temporal uncertainty in the data. The visualizations do, however, identify artifacts from the environmental toxicity model that may introduce noise into our measures in a geographically systematic way.

This approach is an intermediate step for any space-based heath outcome research as it demonstrates the extent of uncertainty about the placement of a subject in some context space. The magnitude of the uncertainty and the overall goals of the project dictate how to proceed. In the example presented here, our concern is placing children in the RSEI space. We might, for example, run subsequent analyses on a subset of FLP points where the PCV, NCV, or LCV is particularly low and see if those points (where uncertainty is lower) produce substantially different results than when we observe all points together. Given relatively low values for NCV we might decide to generalize RSEI model findings from a 0.5 mile (0.805 km) grid to a 2.5 mile (4.023 km) grid–reducing the likelihood that points are assigned to the wrong polygon and limiting the uncertainty associated with PCV. We might choose to assign contextual values that are based on the average across several years of toxicity data to reduce the likelihood that extreme events in the toxicity data are driving outcomes. In an extreme situation, the results might cause a researcher to abandon a dataset altogether.

More broadly, the specific tests presented here are not appropriate for every analytic case. The cases of polygon to polygon or point to point are not considered. Linear data is also highly relevant today with increasing use of personal activity monitors that track a person’s movement through space and time. Our intention here is not to be comprehensive, but to foster a conversation within health and environment research on what the implications of geographic uncertainty might be so that contextual effects can be better captured and understood.

## Data Availability

The point data that support the findings of this study are available from the Family Life Project but restrictions apply to the availability of these data, which were used under license for the current study, and so are not publicly available. The point data are however available from the authors upon reasonable request and with permission of the Family Life Project. The context dataset (RSEI data) analysed during the current study can be generated using the R scripts in the rseilution repository, *https://github.com/dfolch/rseilution*.

## Abbreviations

EPA: US Environmental Protection Agency
FLP: Family Life Project
GIS: Geographic information system
GPS: Global positioning system
LCV: Life-course coefficient of variation
MAUP: Modifiable areal unit problem
MOE: Margin of error
NCV: Neighborhood coefficient of variation
PCV: Positional coefficient of variation
PM: Particulate matter
RSEI: Risk-Screening Environmental Indicators
TRI: Toxic Release Inventory
UGCoP: Uncertain geographic context problem

## Footnotes

1 Although three of the six counties were part of metropolitan statistical areas as defined by the US Office of Management and Budget in June 2003, all six were outside of the large metropolitan concentrations in their state.

## References

[1] Cummins S, Macintyre S. Food environments and obesity—neighbourhood or nation? Int J Epidemiol [Internet]. 2005;35:100–104. Available from: 10.1093/ije/dyi276 10.1093/ije/dyi276 16338945

[2] Inagami S, Cohen DA, Finch BK, Asch SM (2006). You are where you shop: grocery store locations, weight, and neighborhoods. Am J Prev Med [Internet].

[3] Steptoe A, Feldman PJ. Neighborhood problems as sources of chronic stress: development of a measure of neighborhood problems, and associations with socioeconomic status and health. Ann Behav Med [Internet]. 2001;23:177–185. Available from: 10.1207/S15324796ABM2303_5*.* 10.1207/S15324796ABM2303_5 11495218

[4] Stigsdotter UK, Ekholm O, Schipperijn J, Toftager M, Kamper-Jørgensen F, Randrup TB (2010). Health promoting outdoor environments - Associations between green space, and health, health-related quality of life and stress based on a Danish national representative survey. Scand J Public Health [Internet].

[5] Freedman VA, Grafova IB, Rogowski J (2011). Neighborhoods and chronic disease onset in later life. Am J Public Health [Internet].

[6] Eschbach K, Mahnken JD, Goodwin JS. Neighborhood composition and incidence of cancer among Hispanics in the United States. Cancer [Internet]. 2005;103:1036–1044. Available from: 10.1002/cncr.20885 10.1002/cncr.20885 PMC1853250 15672387

[7] Lisabeth L, Diez Roux A, Escobar J, Smith M, Morgenstern L (2006). Neighborhood environment and risk of ischemic stroke: the brain attack surveillance in Corpus Christi (BASIC) project. Am J Epidemiol [Internet].

[8] Chen H, Kwong JC, Copes R, Tu K, Villeneuve PJ, Van Donkelaar A (2017). Living near major roads and the incidence of dementia, Parkinson’s disease, and multiple sclerosis: a population-based cohort study. Lancet.

[9] Couclelis H (2003). The certainty of uncertainty: GIS and the limits of geographic knowledge. Transact GIS [Internet].

[10] Fotheringham A, Wong D (1991). The modifiable areal unit problem in multivariate statistical analysis. Environ Plan A.

[11] Kwan M. How GIS can help address the uncertain geographic context problem in social science research. Ann GIS. 2012.

[12] Fowler CS, Frey N, Folch DC, Nagle N, Spielman S. Who are the people in my neighborhood?: the “contextual fallacy” of measuring individual context with census geographies. Geogr Anal. 2019:155–68.

[13] Robertson C, Feick R. Inference and analysis across spatial supports in the big data era: Uncertain point observations and geographic contexts. Transact GIS [Internet]. 2018; Available from: http://doi.wiley.com/10.1111/tgis.12321.

[14] Kwan M (2012). The uncertain geographic context problem. Ann Assoc Am Geogr.

[15] Openshaw S (1984). Ecological fallacies and the analysis of areal census data. Environ Plan A.

[16] Robinson WS (1950). Ecological correlations and the behavior of individuals. Am Sociol Rev.

[17] Beckx C, Int Panis L, Uljee I, Arentze T, Janssens D, Wets G. Disaggregation of nation-wide dynamic population exposure estimates in The Netherlands: Applications of activity-based transport models. Atmos Environ [Internet]. 2009 [cited 2021 Jan 11];43:5454–5462. Available from: http://www.sciencedirect.com/science/article/pii/S1352231009006311

[18] Dhondt S, Beckx C, Degraeuwe B, Lefebvre W, Kochan B, Bellemans T, et al. Health impact assessment of air pollution using a dynamic exposure profile: Implications for exposure and health impact estimates. Environ Impact Assess Rev [Internet]. 2012 [cited 2021 Jan 11];36:42–51. Available from: http://www.sciencedirect.com/science/article/pii/S0195925512000315

[19] Dhondt S, Beckx C, Degraeuwe B, Lefebvre W, Kochan B, Bellemans T, et al. Integration of population mobility in the evaluation of air quality measures on local and regional scales. Atmos Environ [Internet]. 2012 [cited 2021 Jan 11];59:67–74. Available from: http://www.sciencedirect.com/science/article/pii/S135223101200427X

[20] Gauderman WJ, Avol E, Lurmann F, Kuenzli N, Gilliland F, Peters J, et al. Childhood Asthma and Exposure to Traffic and Nitrogen Dioxide. Epidemiology [Internet]. 2005 [cited 2021 Jan 11];16:737–743. Available from: https://journals.lww.com/epidem/Fulltext/2005/11000/Childhood_Asthma_and_Exposure_to_Traffic_and.5.aspx 10.1097/01.ede.0000181308.51440.75 16222162

[21] Hatzopoulou M, Miller EJ. Linking an activity-based travel demand model with traffic emission and dispersion models: Transport’s contribution to air pollution in Toronto. Transp Res Part D: [Internet]. 2010 [cited 2021 Jan 11];15:315–325. Available from: http://www.sciencedirect.com/science/article/pii/S1361920910000386

[22] Panis LI. New Directions: Air pollution epidemiology can benefit from activity-based models. Atmos Environ [Internet]. 2010 [cited 2021 Jan 11];44:1003–1004. Available from: http://www.sciencedirect.com/science/article/pii/S1352231009009340

[23] Cayo MR, Talbot TO. Positional error in automated geocoding of residential addresses. Int J Health Geogr [Internet]. 2003 [cited 2021 Jan 11];2:10. Available from: 10.1186/1476-072X-2-10 10.1186/1476-072X-2-10 PMC324564 14687425

[24] Lane KJ, Kangsen Scammell M, Levy JI, Fuller CH, Parambi R, Zamore W, et al. Positional error and time-activity patterns in near-highway proximity studies: An exposure misclassification analysis. Environ Health [Internet]. 2013 [cited 2021 Jan 11];12:75. Available from: 10.1186/1476-069X-12-75 10.1186/1476-069X-12-75 PMC3907019 24010639

[25] Schootman M, Sterling DA, Struthers J, Yan Y, Laboube T, Emo B, et al. Positional Accuracy and Geographic Bias of Four Methods of Geocoding in Epidemiologic Research. Ann Epidemiol [Internet]. 2007 [cited 2021 Jan 11];17:464–470. Available from: http://www.sciencedirect.com/science/article/pii/S1047279707000592 10.1016/j.annepidem.2006.10.015 17448683

[26] Zandbergen PA. Influence of geocoding quality on environmental exposure assessment of children living near high traffic roads. BMC Public Health [Internet]. 2007 [cited 2021 Jan 11];7:37. Available from: 10.1186/1471-2458-7-37*.* 10.1186/1471-2458-7-37 PMC1838415 17367533

[27] Zandbergen Paul A., Green Joseph W. Error and Bias in Determining Exposure Potential of Children at School Locations Using Proximity-Based GIS Techniques. Environl Health Perspect [Internet]. 2007 [cited 2021 Jan 11];115:1363–1370. Available from: https://ehp.niehs.nih.gov/doi/10.1289/ehp.9668 10.1289/ehp.9668 PMC1964899 17805429

[28] Ortega Garcia JA, Lopez Hernandez FA, Carceles Alvarez A, Fuster-Soler JL, Sotomayor DI, Ramis R. Childhood cancer in small geographical areas and proximity to air-polluting industries. Environ Res [Internet]. 2017 [cited 2021 Jan 11];156:63–73. Available from: http://www.sciencedirect.com/science/article/pii/S0013935116307010 10.1016/j.envres.2017.03.009 PMC5685507 28319819

[29] Reynolds Peggy, Von Behren Julie, Gunier Robert B, Goldberg Debbie E, Hertz Andrew, Smith Daniel F. Childhood cancer incidence rates and hazardous air pollutants in California: An exploratory analysis. Environ Health Perspect [Internet]. 2003 [cited 2021 Jan 11];111:663–668. Available from: https://ehp.niehs.nih.gov/doi/10.1289/ehp.5986 10.1289/ehp.5986 PMC1241461 12676632

[30] Vernon-Feagans L, Cox M, Willoughby M, Burchinal M, Garrett-Peters P, Mills-Koonce R, et al. The Family Life Project: An epidemiological and developmental study of young children living in poor rural communities. Monogr Soc Res Child Dev. 2013;i–150. 10.1111/mono.12046 24147448

[31] Environmental Protection Agency. Find Out What’s Happening in Your Neighborhood: Using EPA’s Toxics Release Inventory. https://www.epa.gov/sites/production/files/2015-10/documents/2015_tri_for_communities_fact_sheet_final.pdf: Toxics Release Inventory Program; 2015.

[32] Gatzke-Kopp LM, Warkentien S, Willoughby M, Fowler C, Folch DC, Blair C (2021). Proximity to sources of airborne lead is associated with reductions in Children's executive function in the first four years of life. Health Place.

[33] Environmental Protection Agency. EPA’s Risk-Screening Environmental Indicators (RSEI) Methodology. Office of Pollution Prevention; Toxics; 2018. Report No.: RSEI Version 2.3.6.

[34] Ratcliffe JH. On the accuracy of TIGER-type geocoded address data in relation to cadastral and census areal units. Int J Geogr Inf Sci [Internet]. 2001 [cited 2021 Jan 11];15:473–485. Available from: 10.1080/13658810110047221*, On the accuracy of TIGER-type geocoded address data in relation to cadastral and census areal units.*

[35] Galster GC. Making our neighborhoods, making our selves. University of Chicago Press; 2019, DOI: 10.7208/chicago/9780226599991.001.0001.

[36] Kulldorff M, Song C, Gregorio D, Samociuk H, DeChello L (2006). Cancer map patterns: are they random or not?. Am J Prev Med.

[37] Delgado-Saborit JM, Aquilina NJ, Meddings C, Baker S, Harrison RM (2011). Relationship of personal exposure to volatile organic compounds to home, work and fixed site outdoor concentrations. Sci Total Environ.

[38] Burgoine T, Jones AP, Brouwer RJN, Neelon SEB (2015). Associations between BMI and home, school and route environmental exposures estimated using GPS and GIS: do we see evidence of selective daily mobility bias in children?. Int J Health Geogr.

[39] Steinle S, Reis S, Sabel CE. Quantifying human exposure to air pollution-Moving from static monitoring to spatio-temporally resolved personal exposure assessment. Sci Total Environ [Internet]. 2013;443:184–193. Available from: 10.1016/j.scitotenv.2012.10.098 10.1016/j.scitotenv.2012.10.098 23183229

[40] Frei P, Mohler E, Neubauer G, Theis G, Bürgi A, Fröhlich J, Braun-Fahrländer C, Bolte J, Egger M, Röösli M (2009). Temporal and spatial variability of personal exposure to radio frequency electromagnetic fields. Environ Res.

[41] Smith KH, Neilsen TB, Grimshaw J. Full-day noise exposure for student musicians at Brigham Young University. Proceedings of Meetings on Acoustics. Acoust Soc Am. 2017:1–11.

[42] Burke LE, Shiffman S, Music E, Styn MA, Kriska A, Smailagic A, Siewiorek D, Ewing LJ, Chasens E, French B, Mancino J, Mendez D, Strollo P, Rathbun SL (2017). Ecological momentary assessment in behavioral research: addressing technological and human participant challenges. J Med Internet Res.

[43] Spielman SE, Folch DC, Nagle NN (2014). Patterns and causes of uncertainty in the American community survey. Appl Geogr.

[44] Folch DC, Arribas-Bel D, Koschinsky J, Spielman SE (2016). Spatial variation in the quality of American community survey estimates. Demography..

